# Three new species of *Inosperma* (Agaricales, Inocybaceae) from Tropical Africa

**DOI:** 10.3897/mycokeys.77.60084

**Published:** 2021-01-28

**Authors:** Hyppolite L. Aïgnon, Sana Jabeen, Arooj Naseer, Nourou S. Yorou, Martin Ryberg

**Affiliations:** 1 Research Unit Tropical Mycology and Plant-Soil Fungi Interactions, Faculty of Agronomy, University of Parakou, 03 BP 125, Parakou, Benin University of Parakou Parakou Benin; 2 Department of Botany, Division of Science and Technology, University of Education, Lahore, Pakistan University of Education Lahore Pakistan; 3 Department of Botany, University of the Punjab, Quaid-e-Azam Campus-54590, Lahore, Pakistan University of the Punjab Lahore Pakistan; 4 Systematic Biology Programme, Department of Organismal Biology, Uppsala University, Norbyvägen 18D, 752 36, Uppsala, Sweden Uppsala University Uppsala Sweden

**Keywords:** Ectomycorrhizal, molecular systematics, phylogeny, taxonomy, West Africa

## Abstract

Here, we describe three new species of *Inosperma* from Tropical Africa: *Inosperma africanum*, *I. bulbomarginatum* and *I. flavobrunneum*. Morphological and molecular data show that these species have not been described before, hence need to be described as new. The phylogenetic placements of these species were inferred, based on molecular evidence from sequences of 28S and RPB2. Additional analysis using ITS dataset shows interspecific variation between each species. Phylogenetic analyses resolve *I. flavobrunneum* in Old World Tropical clade 1 with weak support, *I. bulbomarginatum* is sister of Old World Tropical clade 1 and *I. africanum* is indicated as sister to the rest of *Inosperma*. Complete description and illustrations, including photographs and line drawings, are presented for each species. A new combination of *Inocybe shawarensis* into *Inosperma* is also proposed.

## Introduction

Inocybaceae Jülich (Basidiomycota, Agaricales) is a family of ectomycorrhizal species, forming symbiotic association with more than 23 families of vascular plants (Matheny et al. 2020). The family is diverse with an estimated 1050 species distributed worldwide (Matheny and Kudzma 2019; Matheny et al. 2020). The number of species described will continue to increase as new habitats are explored (Matheny and Watling 2004; Esteve-Raventós 2014; Latha and Manimohan 2015, 2016; Matheny et al. 2017; Naseer et al. 2018; Jabeen and Khalid 2020).

Recently, Inocybaceae was revised to include seven genera, *Auritella* Matheny & Bougher, *Inocybe* (Fr.) Fr., *Inosperma* (Kühner) Matheny & Esteve-Rav., *Mallocybe* (Kuyper) Matheny, Vizzini & Esteve-Rav., *Nothocybe* Matheny & K.P.D. Latha, *Pseudosperma* Matheny & Esteve-Rav. and *Tubariomyces* Esteve-Rav. & Matheny (Matheny et al. 2020). *Inosperma* is represented by more than 70 known species that are distributed in Africa, Asia, Australasia, Europe, North America and northern South America (Matheny et al. 2020). Typically, the species of the genus are characterised by a radially fibrillose and rimose or squamulose pileus; smooth, ellipsoid or phaseoliform basidiospores; and absence of metuloid hymenial cystidia. In addition, many species of *Inosperma* have odours that are fruity, pleasant, like honey, fishy, pelargonium or otherwise distinct (Matheny et al. 2020). Phylogenetically the genus is monophyletic with four major clades: the Maculata clade (Larsson et al. 2009), I. sect. Inosperma and two clades from the Old World tropics (Pradeep et al. 2016; Matheny et al. 2020).

In this study, we describe three new species of *Inosperma* from West Africa, based on morphological characters, as well as analysing their phylogenetic position using multigene molecular analysis of 28S and RPB2 sequences data.

## Material and methods

### Study area and specimen sampling

Specimens were collected in Benin in Okpara Forest (9°15.13'N, 2°43.05'E), N’dali Forest Reserve (09°45.73'N, 2°19.93'E), Toui-Kilibo Forest Reserve (8°32.74'N, 2°40.42'E) and Alibori Superieur Forest Reserve (10°23.76'N, 2°5.15'E). Additionally, specimens were collected in, Burkina Faso in the Forest Reserve of Kou (10°55.86'N,4°51.83'W); Ivory Coast in Gbeke Region (7°40.52'N, 4°54.48'W), Guinea in National Park of Haut Niger (10°30.76'N, 9°57.68'W) and Togo in Central Region (09°20.38'N, 1°14.44'E).

The habitats are woodland dominated by *Isoberlinia doka* Craib & Stapf, *I. tomentosa* (Harms) Craib et Stapf, *Uapaca togoensis* Pax or gallery forest dominated by *Berlinia grandiflora* (Vahl) Hutch. Specimens were preserved by drying on an electric dryer (type Stöckli Dörrex) for 24 hours at 45 °C. All studied materials are deposited at the Mycological Herbarium of Parakou University (UNIPAR).

### Morphological analyses

Specimens were photographed in the field with a digital camera Sony FE. Colour codes are described using Kornerup and Wanscher (1978). For anatomical analyses, samples

of specimens were rehydrated and examined directly in 3% potassium hydroxide (KOH) and Congo red. Drawings of microscopic characters were made with the aid of a drawing tube attached to a Leica DM2700. Microscopic characters were drawn at magnification 1000×. Spore measurements were made from 40 spores for each species. We measured length (L) and width (W) of the basidiospores and calculated the ratio Q = L / W. Measurements of basidiospores and basidia excluded the apiculus and sterigmata, respectively and are given as (a–)b–c(–d), where (a) = extreme minimum value, range b–c contains minimum of 90% of the calculated values and (d) = extreme maximum value as used in Aïgnon et al. (2021).

### Molecular analyses

#### DNA extraction, PCR and sequencing

Genomic DNA was extracted from dried specimens by QIAGEN® plant mini kit following the manufacturer’s instructions and PCR products were cleaned using ExoSAP-IT (Bell 2018). The internal transcribed spacer regions (ITS), portions of the nuclear large subunit ribosomal RNA gene (28S) and DNA-directed RNA polymerase II subunit (RPB2) were amplified. For sequencing of the ITS region, we used the primers ITS1F and ITS4 (White et al. 1990; Gardes and Bruns 1993), for LSU we used LR0R, LR7 and internal primers LR5 and LR3R (Vilgalys and Hester 1990; Cubeta et al. 1991; Rehner and Samuels 1995) and for RPB2, we used primer pairs b6F and b7.1R (Matheny 2005). PCR products were cleaned and sequenced at Macrogen Inc. (Macrogen Europe B.V., Amsterdam, Netherlands) using the same primers as those used for PCR.

#### Sequence alignments and phylogenetic analyses

Nineteen new sequences were generated (Table 1). Sequences were BLAST searched against NCBI and similar sequences were retrieved from GenBank (Benson et al. 2010). The sequences of ITS, 28S and RPB2 were aligned separately in MAFFT V7.464 (Katoh et al. 2019). Alignment is available online in TreeBase under accession number 27445 (http://purl.org/phylo/treebase/phylows/study/TB2:S27445).

For phylogenetic analysis, the dataset of 28S and RPB2 was generated using Geneious 7.0.2 (Drummond et al. 2010) and partitioned in 28S, RPB2 codon position 1, RPB2 codon position 2, RPB2 codon position 3 and the intron in RPB2 separately (Suppl. material 1). We tested for the best partitioning scheme and best model for each partition using Modelfinder (Kalyaanamoorthy et al. 2017). It was indicated that keeping all the partitions was the best way to proceed. Maximum Likelihood (ML) analysis was performed with IQTREE 1.6.12 (Nguyen et al. 2015). Branch support was assessed with 1000 replicates of ultrafast bootstrap replicates and approximate likelihood ratio test [aLRT] and Shimodaira-Hasegawa [SH]-aLRT (SH-Alrt) test with 1000 replicates (Hoang et al. 2017).

For Bayesian Inference (BI) analyses, GTR models with gamma-distributed rate heterogeneity and a proportion of invariant sites parameter were assigned to each partition as indicated above, using MrBayes 3.2.7 (Ronquist et al. 2012), set as follows: lset applyto = (all), nst = 6, rates = invgamma, ngammacat = 4, sampling frequency = 1000 and the command “unlink” was used to unlink parameters across characters on partitioned datasets. Two independent Markov Chain Monte Carlo (MCMC) processes were executed, each in four chains for 20 million generations. Posterior probabilities (BPP) were calculated after burning the first 25% of the posterior sample and ensuring that this threshold met the convergence factors described above. The sequences from *Pseudosperma lepidotellum* (Matheny & Aime) Matheny & Esteve-Rav., *P. pluviorum* (Matheny & Bougher) Matheny & Esteve-Rav., *Pseudosperma* sp. PBM3751 and *Pseudosperma* sp. TR194-02 were used as outgroup taxa. We also produced trees using ITS database only to show interspecific variation between each species.

**Table 1.** List of species, geographic origin and GenBank accession numbers of ITS, 28S and RPB2 sequences used in the molecular analysis; the new species and new combinations are in bold.

Species	Voucher	Country	ITS	28S	RPB2	References
*Auritella brunnescens* Matheny & Bougher	PBM3174	Australia	KJ702344	JQ313571	KJ702349	Matheny et al. (2017)
*Auritella dolichocystis* Matheny, Trappe & Bougher	Trappe 24844	New South Wales		AY380371	AY337371	Matheny (2005)
*Auritella fulvella* Matheny & Bougher	BRI:AQ669485	Australia	KJ702355	KJ702353	KJ702357	Matheny et al. (2017)
*Auritella hispida* Matheny & T.W. Henkel	TH1009, TH10379	Cameroon	KT378203	KT378208	KT378215	Matheny et al. (2017)
*Auritella serpentinocystis* Matheny, Trappe &Bougher ex Matheny & Bougher	PBM3188	Australia	KJ729858	JQ313559	KJ756402	Matheny et al. (2017)
*Auritella spiculosa* Matheny & T.W. Henkel	MCA7031, TH9866	Cameroon	MF374763	KT378206	KT378214	Matheny et al. (2017)
*Inosperma adaequatum* (Britzelm.) Matheny & Esteve-Rav.	JV 16501F, JV11290F	Finland	JQ801381	JQ815407	AY333771	Matheny et al. (2020)
***I. africanum* Aïgnon, Yorou & Ryberg**	**MR00387**	**Togo**	**MN096189**	**MN097881**	**MT770739**	**This study**
	**HLA0361**	**Benin**	**MT534295**	**MT560735**		
	**HLA0383**	**Benin**	**MT534298**	**MT560733**		
	**HLA0353**	**Benin**	**MT534299**			
	BRF4157	Benin		MK908843		Unpublished
*I. akirnum* (K.P.D. Latha & Manimohan) Matheny & Esteve-Rav.	CAL 1358	India		NG_057279	KY553236	Latha and Manimohan (2016)
*I. apiosmotum* (Grund& D.E. Stuntz) Matheny & Esteve-Rav.	AU10560, TENN:062779	Canada, USA	HQ201336	JN975022	JQ846463	Ryberg and Matheny (2012)
*I. bongardii* (Weinm.) Matheny & Esteve-Rav.	EL9406	Sweden	FN550943	FN550943		Unpublished
***I. bulbomarginatum* Aïgnon, Yorou & Ryberg**	**MR00357**	**Benin**	**MN096190**	**MN097882**	**MN200775**	**This study**
	**HLA0373**	**Benin**	**MT534301**			
	**HLA0389**	**Benin**	**MT534302**			
	**HLA0417**	**Benin**	**MT534300**	**MT560734**		
	PC96082	Zambia	JQ801412	JN975027		Ryberg and Matheny (2012)
*I. calamistratoides* (E. Horak) Matheny & Esteve-Rav.	PBM3384	Australia		JQ815415	KJ729949	Latha and Manimohan (2016)
*I. calamistratum* (Fr.) Matheny & Esteve-Rav.	PBM1105	USA	JQ801386	JQ815409	JQ846466	Pradeep et al. (2016)
*I. carnosibulbosum* (C.K. Pradeep & Matheny) Matheny & Esteve-Rav.	TBGT:12047	India	KT329448	KT329454	KT32944	Pradeep et al. (2016)
*I. cervicolor* (Pers.) Matheny & Esteve-Rav.	SJ04024, TURA:4761	Sweden, Finland	AM882939	AM882939	JQ846474	Ryberg et al. (2008)
*I. cookei* (Bres.) Matheny & Esteve-Rav.	EL70A03	Sweden	AM882953	AM882953		Ryberg et al. (2008)
*I. cyanotrichium* (Matheny, Bougher& G.M. Gates) Matheny & Esteve-Rav	TENN:065729	Australia		JQ815418	KJ729948	Unpublished
***I. flavobrunneum* Aïgnon, Yorou & Ryberg**	**HLA0367**	**Benin**	**MN096199**	**MT536754**		**This study**
	**HLA0372**	**Benin**	**MT534290**	**MT536756**		
*I. geraniodorum* (J. Favre) Matheny & Esteve-Rav.	EL10606	Sweden	FN550945	FN550945		Latha and Manimohan (2016)
*I. gregarium* (K.P.D. Latha & Manimohan) Matheny & Esteve-Rav.	CAL 1309	India	KX852305	KX852306	KX852307	Latha and Manimohan (2016)
*I. lanatodiscum* (Kauffman) Matheny & Esteve-Rav.	PBM2451	USA	JQ408759	JQ319688	JQ846483	Latha and Manimohan (2016)
*I. maculatum* (Boud.) Matheny & Esteve-Rav.	MR00020	Sweden	AM882958	AM882958		Ryberg et al. (2008)
*I. maximum* (A.H. Sm.) Matheny & Esteve-Rav.	PBM 2222,UBC F33244	USA,Canada	MG953983	EU569854		Matheny et al. (2009)
*I. misakaense* (Matheny & Watling) Matheny &Esteve-Rav.	96234 (PC)	Zambia	JQ801409	EU569874	EU569873	Pradeep et al. (2016)
*I. mutatum* (Peck) Matheny & Esteve-Rav.	PBM4108, PBM2953	USA	MG773837	JQ994476	JQ846488	Matheny et al. (2020)
*I. neobrunnescens* (Grund & D.E. Stuntz) Matheny &Esteve-Rav.	PBM 2452	USA		EU569868	EU569867	Matheny et al. (2009)
*I. quietiodor* (Bon) Matheny & Esteve-Rav.	PAM01091310	France	FJ936168	FJ936168		Larsson et al. (2009)
*I. rhodiolum* (Bres.) Matheny & Esteve-Rav.	PAM00090117	France	FJ904176	FJ904176		Larsson et al. (2009)
*I. rimosoides* (Peck) Matheny & Esteve-Rav.	PBM 2459, PBM3311	USA	JQ801414	JQ815426	DQ385884	Latha and Manimohan (2016)
*I. rubricosum* (Matheny & Bougher) Matheny & Esteve-Rav.	PBM3784	Australia		NG_057260	KM406230	Horak et al. (2015)
***I. shawarense* (Naseer & Khalid) Aïgnon & Naseer**	**FLAS-FS9456**	**Pakistan**	**KY616965**	**KY616966**		**Naseer et al. (2018)**
*Inosperma* sp.	DB166	Democratic Republic of the Congo	KT461385			Bauman et al. (2016)
*Inosperma* sp.	PC 96013	Zambia	JQ801383	JQ815408	EU600882	Matheny et al. (2009)
*Inosperma* sp.	BB3233	Zambia	JQ801415	EU600885		Matheny et al.(2009)
*Inosperma* sp.	G1842	Zambia		MK278245		Unpublished
*Inosperma* sp.	TR220_06	Papua New Guinea	JQ801416	JN975017	JQ846496	Ryberg and Matheny (2012)
*Inosperma* sp.	L-GN3a	Papua New Guinea	JX316732			Tedersoo and Põlme (2012)
*Inosperma* sp.	Zam07	Zambia	FR731653			Tedersoo et al. (2011)
*Inosperma* sp.	PBM3406	Australia		JQ815431	JQ846498	Unpublished
*Inosperma* sp.	TJB10045	Thailand	KT600658	KT600659	KT600660	Pradeep et al. (2016)
*Inosperma* sp.	PC 96073	Zambia	JQ801417	EU600870	EU600869	Matheny et al. (2009)
*Inosperma* sp.	PC:96080	Zambia	JQ801382			Unpublished
*I. vinaceobrunneum* (Matheny, Ovrebo & Kudzma) Haelew.	TENN:062709, PBM 2951	USA	FJ601813	NG_067775	JQ846478	Matheny and Kudzma (2019)
*I. viridipes* (Matheny, Bougher & G.M. Gates) Matheny & Esteve-Rav.	PBM3767	Australia	NR_153168	KP171094	KM656138	Latha and Manimohan (2016)
*I. virosum* (K.B. Vrinda, C.K. Pradeep, A.V. Joseph & T.K. Abraham ex C.K. Pradeep, K.B. Vrinda& Matheny) Matheny & Esteve-Rav.	TBGT:753	India	KT329452	KT329458	KT329446	Pradeep et al. (2016)
*Mallocybe myriadophylla* (Vauras & E. Larss.) Matheny & Esteve-Rav.	JV19652F	Finland	DQ221106	AY700196	AY803751	Matheny et al. (2007)
*M. subdecurrens* (Ellis & Everh.) Matheny & Esteve-Rav.	REH10168	USA	MH024850	MH024886	MH577503	Matheny et al. (2020)
*M. terrigena* (Fr.) Matheny, Vizzini & Esteve-Rav.	EL11704, JV 16431	Sweden	AM882864	AY380401	AY333309	Matheny and Ammirati (2003); Matheny (2005)
*M. tomentosula* Matheny & Esteve-Rav.	PBM4138	USA	MG773814	MK421969	MH577506	Matheny et al. (2020)
*M. unicolor* (Peck) Matheny & Esteve-Rav.	PBM 1481	USA		AY380403	AY337409	Matheny (2005)
*Pseudosperma*lepidotellum (Matheny & Aime) Matheny & Esteve-Rav.	TENN066442	Guyana	JN642233	NG_042597	MH577508	Matheny et al. (2012)
*P. pluviorum* (Matheny & Bougher) Matheny & Esteve-Rav.	BRI:AQ794010, PERTH:08556466	Australia		NG_057259	KM406221	Horak et al. (2015)
*Pseudosperma* sp.	PBM3751	Australia	KP636851	KP171053	KM555145	Pradeep et al. (2016)
*Pseudosperma* sp.	TR194-02 (M)	Papua New Guinea	JQ408793	JN975032	JQ421080	Ryberg and Matheny (2012)
*Tubariomyces inexpectatus* (M. Villarreal, Esteve-Rav., Heykoop & E. Horak) Esteve-Rav. & Matheny	AH25500 AH20390	Spain	GU907095	EU569855	GU907088	Matheny et al. (2009), Alvarado et al. (2010)
*T. similis* Della Magg., Tolaini & Vizzini	RFS0805	Spain	GU907096	GU907092	GU907089	Alvarado et al. (2010)
*T. hygrophoroides* Esteve-Rav., P.-A. Moreau & C.E. Hermos.	P05112008	France	GU907097	GU907094	GU907090	Pradeep et al. (2016)

## Results

### Phylogenetic analyses

*Inosperma* is indicated as monophyletic with full bootstrap support. All three of the species described here, *Inosperma africanumI. bulbomarginatum* and *I. flavobrunneum*, are members of this genus. Phylogenetically, *I. africanum* is indicated as sister to the rest of *Inosperma*, with full support (99.9% SH-aLRT values, 100% ML ultrafast bootstrap, 1 BPP). The Old World Tropical clade 1 is retrieved with strong support (93.8% SH-aLRT values, 99% ML bootstrap, 1 BPP) and *I. bulbomarginatum* is indicated as the sister of Old World Tropical clade 1 with full bootstrap support (100% SH-aLRT values, 100% ML Ultrafast bootstrap, 1 BPP). The sequences of collection PC96082 are very similar to the sequences of *I. bulbomarginata* that we infer to be of the same species. *Inosperma flavobrunneum* is nested in Old World Tropical clade 1 as sister species to three undescribed collections, BB3233, G1842 and PC96013, all from Zambia with weak support.

### Taxonomy

#### 1. Inosperma africanum

Taxon classificationFungiAgaricalesInocybaceae

Aïgnon, Yorou & Ryberg sp. nov.

DD5D33D0-4E7C-5512-8885-7B3A787BF651

836199

Figs 2a , 3

##### Diagnosis.

*Inosperma africanum* is distinct from all species of *Inosperma* and truly outstanding by its vinaceous to red colouration.

##### Type.

***Holotype*.** Benin, Collines Region, Kilibo: 8°32.74'N, 2°40.42'E, on soil in Forest Reserve of Toui-Kilibo in Woodland dominated by *Isoberlina doka* and *I. tomentosa*, 11 August 2017, leg. AIGNON L.H, Voucher (HLA0383) GenBank accession: ITS (MN096193); LSU (MN097885) and RPB2 (MT770739).

##### Description.

Pileus 8.5–15 mm diam., convex to plane, uniform, surface fibrillose, vinaceous to red (8F8), surface rimose, dry. Lamellae moderately close, subventricose, narrowly attached, 0.5–1 mm deep; vinaceous, sometimes light pinkish (8F8), edges fimbriate, vinaceous (8B8). Stipe 15–23 × 0.5–1 mm, cylindrical, central, fibrillose, swollen, bulbous at the base, veil none with the lower two thirds pinkish-white (8A3) and the upper third light vinaceous (8A5). Odour and taste not distinctive. Basidiospores (6.2) 8–10 (10.3) × (3.8) 4–6.8 (7) μm, avl × avw = 8.3 × 5.3 μm, Q = (1.2) 1.1–2.1 (2.2), avQ = 1.6, smooth, (sub) globose to cylindrical, sometimes ellipsoid. Basidia 18–47 × 7–10 μm, clavate, 3–4 sterigmate, hyaline. Cheilocystidia 22–54 × 8–12 μm, cylindrical to clavate, thin-walled, hyaline. Pleurocystidia absent. Pileipellis a cutis with cylindrical, smooth, thin-walled hyphae, 6–20 μm diam., negative reaction of pileus surface in KOH. Stipitipellis a cutis radially arranged, hyphae 5–13 μm diam., parallel, sometimes septate, filamentous. Caulocystidia 22–63 × 8–13 μm, fusiform sometimes utriform, observed on the upper third of the stipe. Clamp connections present.

##### Distribution.

Currently known from Benin, Burkina Faso, Guinea, Ivory Coast, Togo.

##### Ecology.

Scattered in Tropical Woodlands dominated by *Isoberlinia doka* and *I. tomentosa* or gallery forests dominated by *Berlina grandiflora*.

##### Etymology.

*africanum*, referring to the distribution in Africa.

##### Additional specimens examined.

Benin, Borgou Province, N’dali Region: 8°32.74'N, 2°40.42'E, on soil in Woodland dominated by *Isoberlina doka*, 30 August 2017 in Forest Reserve of N’dali, Leg. Aïgnon HL., Voucher (HLA0461) GenBank accession: ITS (MT534297) and LSU (MT560732). Benin, Borgou Province, Tchaorou Region: 9°15.28'N, 2°43.38'E, on soil in forest of Okpara in woodland dominated by *I. doka*, 7 June 2017, leg. Aïgnon HL., Voucher (HLA0353) GenBank accession: ITS (MT534299). Benin, Borgou Province, N’dali Region: 8°45.73'N, 2°19.93'E, on soil in Woodland dominated by *Isoberlina doka*, 8 July 2013, leg. Ryberg M., Voucher (MR00361). Benin, Province, Boukoumbe, North Region: 10°14.45'N, 1°7.00'E, on soil in Woodland dominated by *Isoberlina doka*, 25 July 2020 in Koussoukouangou waterfall, Leg. Aïgnon HL., Voucher (HLA0736). Burkina Faso, Kenedougou Province, Toussiambandougou Region: 10°55.86'N, 4°51.83'W, on soil in gallery forest dominated by *Berlina grandiflora*, 27 June 2018, leg. Aïgnon HL., Voucher (HLA0353). Ivory Coast, Kekrekouakoukro Province, Bouake, Gbeke Region: 7°40.52'N, 4°54.48'W, on soil in Woodland dominated by *B. grandiflora*, 11 July 2018, leg. Aïgnon HL., Voucher (HLA0562). Guinea, Faranah Province, Upper Guinea Region, National Park of Haut Niger: 10°30.76'N, 9°57.68'W, on soil in Woodland dominated by *B. grandiflora*, 4 July 2018, leg. Aïgnon HL., Voucher (HLA0532). Togo, Central Region, Prefecture of Assoli, on the road between Bafilo and Aledjo: 09°20.38'N, 1°14.44'E in Woodlands dominated by *I. tomentosa*, 7 August 2013, leg. Martin Ryberg, Voucher (MR00387) GenBank accession: ITS (MN096189); LSU (MN097881), RPB2 (MT770739).

##### Notes.

*Inosperma africanum* is nested in *Inosperma* and indicated as sister to the rest of the genus in our phylogenetic analyses and is very distinct by its small size and a vinaceous to red pileus. It has a wide distribution in West Africa.

#### 2. Inosperma bulbomarginatum

Taxon classificationFungiAgaricalesInocybaceae

Aïgnon, Yorou & Ryberg sp. nov.

FC84E953-A7CF-5FF5-88C3-C078EAEFFD17

836198

Figs 2b , 4

##### Diagnosis.

*Inosperma bulbomarginatum* differs from *I. flavobrunneum* by the smaller size of its basidiomata and larger basidiospores. It is phylogenetically distinct from all other undescribed African *Inosperma* in Old World Tropical clade 2

##### Type.

***Holotype*.** Benin, Borgou Province, N’dali Region: 09°45.73'N, 2°19.93'E, on soil in Woodland dominated by *Isoberlina doka* and *I. tomentosa*, 8 July 2013, leg. Martin Ryberg, Voucher (MR00357), GenBank accession: ITS (MN096190); LSU (MN097882) and RPB2 (MN200775).

##### Description.

Pileus 13–18 mm diam., undulating plane, fibrillose, margin rimose, orange-brown to somewhat cinnamon, greyish-white (8E5), splitting at edge. Lamellae 2–2.5 mm deep, moderately close, narrowly attached, pale grey brown (9B5) to dark brown (9D5), sinuate. Stipe 10–22 × 2–2.5 mm, central, equal, marginate bulb, white to pinkish-buff (7A2), velar remnants. Odour and taste not distinctive. Basidiospores (7.1) 8–12.1 (14) × (4) 4.2–6.7(7) μm, avl × avw = 9.6 × 5.4 μm, Q = (1.3) 1.2–2.3(2.6), avQ = 1.8, smooth, elongate, thick-walled. Basidia (25) 28–40 × 6–12 μm, tetrasporic. Cheilocystidia 20–25 × 10–12 μm, clavate, thin-walled hyaline. Pleurocystidia absent. Pileipellis a cutis, thin-walled hyphae, 3–12 μm diam., cylindrical. Stipitipellis a cutis with subparallel hyphae 3–15 μm diam., septate, filamentous, subhymenium of compact hyphae, any reaction of pileus surface in KOH not observed. Caulocystidia 25–60 × 7–20 μm, ovoid to obovoid, sometimes utriform, observed on the upper third of the stipe.

##### Distribution.

Currently known from Benin and Zambia.

##### Ecology.

Scattered in Woodland dominated by *Isoberlinia doka* and *I. tomentosa*.

##### Etymology.

*bulbomarginatum* referring to the presence of a marginate bulb at the base of the stipe.

##### Additional specimens examined.

Benin, Collines Province, Kilibo Region: 8°32.74'N, 2°40.42'E, on soil in Woodland dominated by *Isoberlina doka*, 22 June 2017 in the Forest Reserve of Toui-Kilibo, leg. Aïgnon HL., Voucher (HLA0389) GenBank accession: ITS (MT534302). Benin, Tchaorou, Borgou Prov, Okpara Forest: 9°15.28'N, 2°43.38'E, on soil in Woodland dominated by *Isoberlina doka*, 13 June 2017, leg. Aïgnon HL., Voucher (HLA0373) GenBank accession: ITS (MT534301). Benin, Alibori Borgou Prov, Alibori Superieur Forest Reserve: 10°23.76'N, 2°5.15'E on soil in Woodland dominated by *Isoberlina doka*, 11 July 2017, in Forest Reserve of Alibori Supérieur leg. Aïgnon HL., Voucher (HLA0417), GenBank accession: ITS (MT534300) and LSU (MT560734).

##### Notes.

*Inosperma bulbomarginatum* is similar to *Inosperma cervicolor* (Pers.) Matheny & Esteve-Rav., by its orange-brown pileus, but differs from it by the smaller size of the basidiomata and basidiospores, as well as its ecological association with Fabaceae Lindley and/or Phyllanthaceae Martynov and extensive distribution in Tropical Africa. *I. cervicolor* is associated with Pinaceae Spreng. ex F. Rudolphi and distributed in Europe and North America.

#### 3. Inosperma flavobrunneum

Taxon classificationFungiAgaricalesInocybaceae

Aïgnon, Yorou & Ryberg sp. nov.

806A46FA-E3C0-5D93-9125-B5DA4D1B748A

836197

Figs 2c, d , 5

##### Diagnosis.

Characterised by yellow to orange-brown pileus, 7–12 × 4–7 μm smooth, thick-walled, ellipsoid basidiospores with cheilocystidia measuring 23–41 × 7–10 μm, clavate, thin-walled.

##### Type.

***Holotype*.** Benin, Borgou Province, Tchaorou, Okpara Forest: 9°15.13'N, 2°43.05'E on soil in Woodland dominated by *Isoberlina doka* 12 June 2017, leg. AIGNON L.H, Voucher (HLA0367), GenBank accession: ITS (MN096199); LSU (MT536754).

##### Description.

Pileus 28–38 mm diam., umbonate, yellow (5A3) to orange brown (5C5), dark brown in middle, convex when young, plane at maturity, hard, surface rimose, dry. Lamellae emarginated, adnexed and decurrent, yellow brown (5B5). Stipe 27–39 × 5–6 mm, central, cylindrical, uniform; white, equal, solid, hard, base slightly swollen to bulbous, pruinose at the apex. Basidiospores (7.1) 9.2–11.2 (12) × (4.1) 5.7–7 (7.2) μm, avl × avw = 9.2 × 5.7 μm, Q = (1.2) 1.6–2.1 (2.5), avQ = 1.6, smooth, ellipsoid. Basidia 24–40 × 6–14 μm, clavate, 2–4 spored. Cheilocystidia 23–41 × 7–10 μm, clavate, thin walled. Pleurocystidia absent. Pileipellis a cutis thin-walled hyphae 4–16 μm diam., subparallel, compact hyphae, negative reaction of pileus surface in KOH. Stipitipellis a cutis hyphae 5–10 μm diam., septate, filamentous, thick, subparallel, compact. Caulocystidia 23–52 × 9–10 μm, utriform, rare, observed on the upper third of the stipe.

##### Distribution.

Currently known only from Benin in Soudano-Guinean zone.

##### Ecology.

Gregarious under Woodland dominated by *Isoberlinia doka*, *I. tomentosa and Monotes kerstingii* Gilg.

##### Etymology.

*flavobrunneum* referring to yellow to dark brown pileus.

##### Additional specimens examined.

Benin, Tchaorou, Borgou Province, Okpara Forest: 9°15.27'N, 2°43.40'E on soil in Woodland dominated by *Isoberlina doka*, *I. tomentosa* 13 June 2017, leg. AIGNON L.H, HLA0372, GenBank accession: ITS (MT534290); LSU (MT536756).

##### Notes.

**Figure 1.**
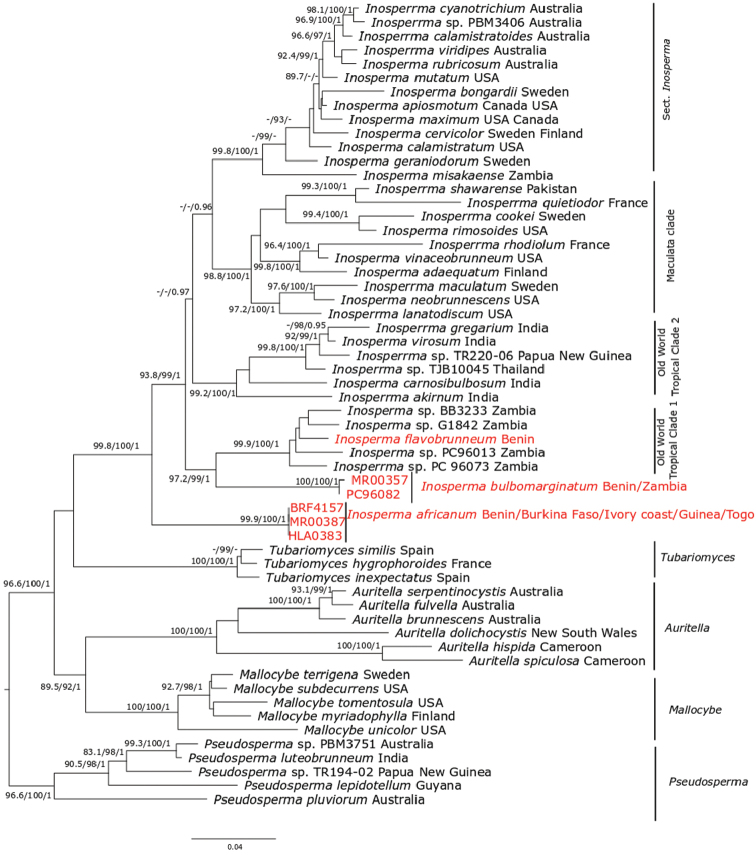
ML tree of 28S and RPB2 sequences showing the placement of *Inosperma africanum*, *I. bulbomarginatum* and *I. flavobrunneum*. Values above or below branches indicate bootstrap proportions SH-aLRT support ≥ 80% / ultrafast bootstrap support ≥ 95% / Bayesian posterior probabilities > 0.95 as shown. Origin of species is given after the name of each taxon. The new species are in red.

In the phylogenetic tree (Figure 1), *Inosperma flavobrunneum* is a sister of *Inosperma* sp. PC96013, an undescribed species from Zambia in Miombo woodland. Morphologically, *I. flavobrunneum* is similar to *I. lanatodiscum* by its yellow to orange-brown pileus, but differs from it by the smaller size of the basidiomata, larger basidiospores, ecological association with Fabaceae / Dipterocarpaceae Blume and distribution in West Africa. *I. lanatodiscum* is associated with a variety of hardwoods and conifers and is widely distributed from Europe to North and Central America (Kropp et al. 2013). The other related taxa are all African taxa not yet described, such as *Inosperma* sp. BB3233 from Zambia and the Democratic Republic of Congo, as well as *Inosperma* sp. G1842 distributed in south-eastern Africa, while *I. flavobrunneum* is distributed in West Africa.

**Figure 2.**
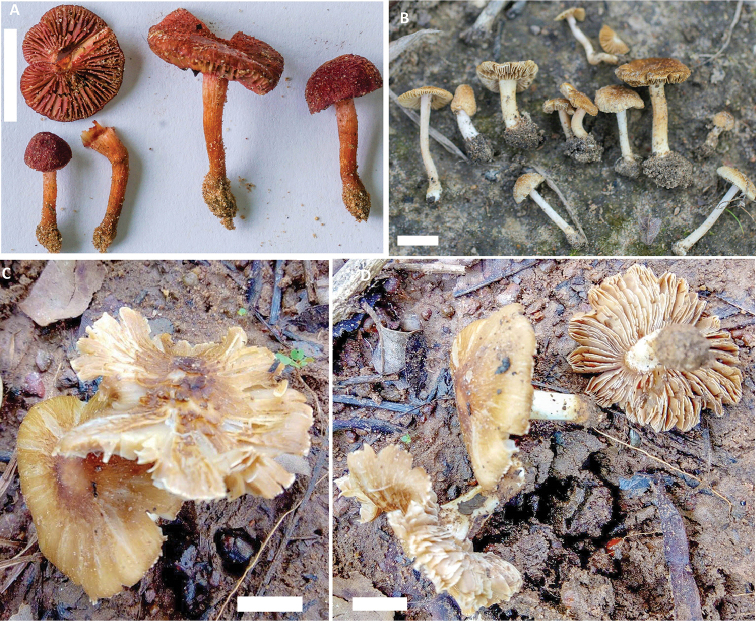
Macromorphology of: **A***Inosperma africanum* (HLA0383) **B***Inosperma bulbomarginatum* (MR00357) **C, D***Inosperma flavobrunneum* (HLA0367). Scale bar: 1 cm.

**Figure 3.**
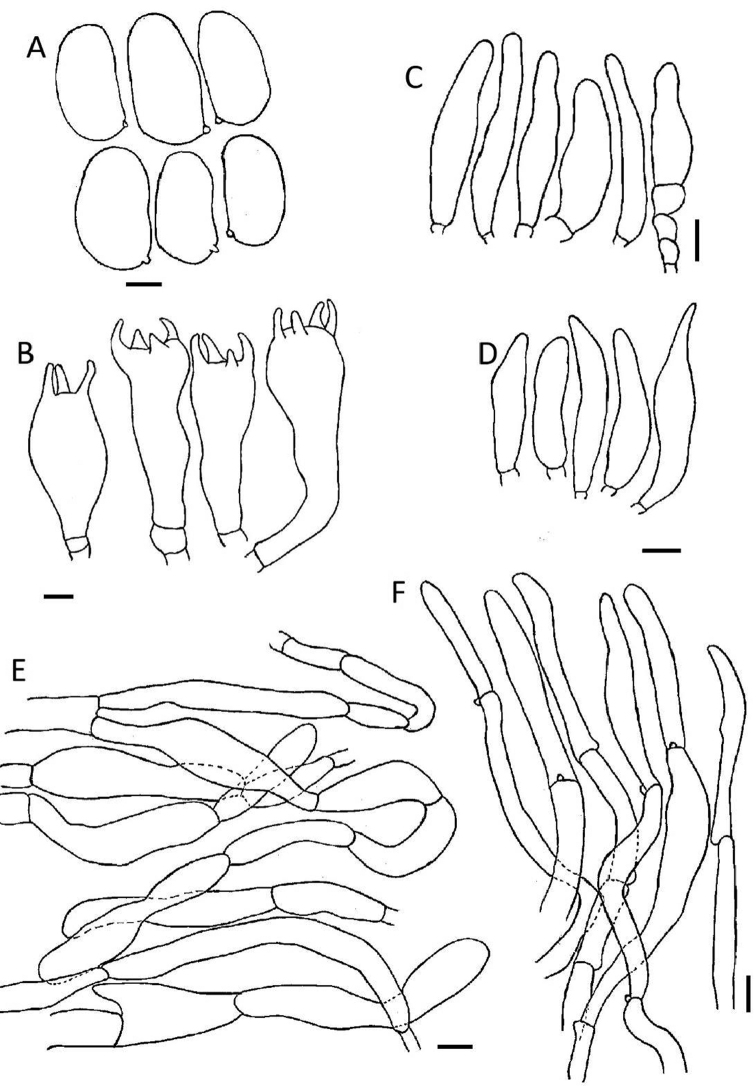
Microscopic structures of *Inosperma africanum* (HLA0383) **A** basidiospores **B** basidia **C** cheilocystidia **D** caulocystidia **E** pileipellis **F** stipitipellis. Scale bars: 3 μm (**A**); 5 μm (**B**); 10 μm (**C–F**).

**Figure 4.**
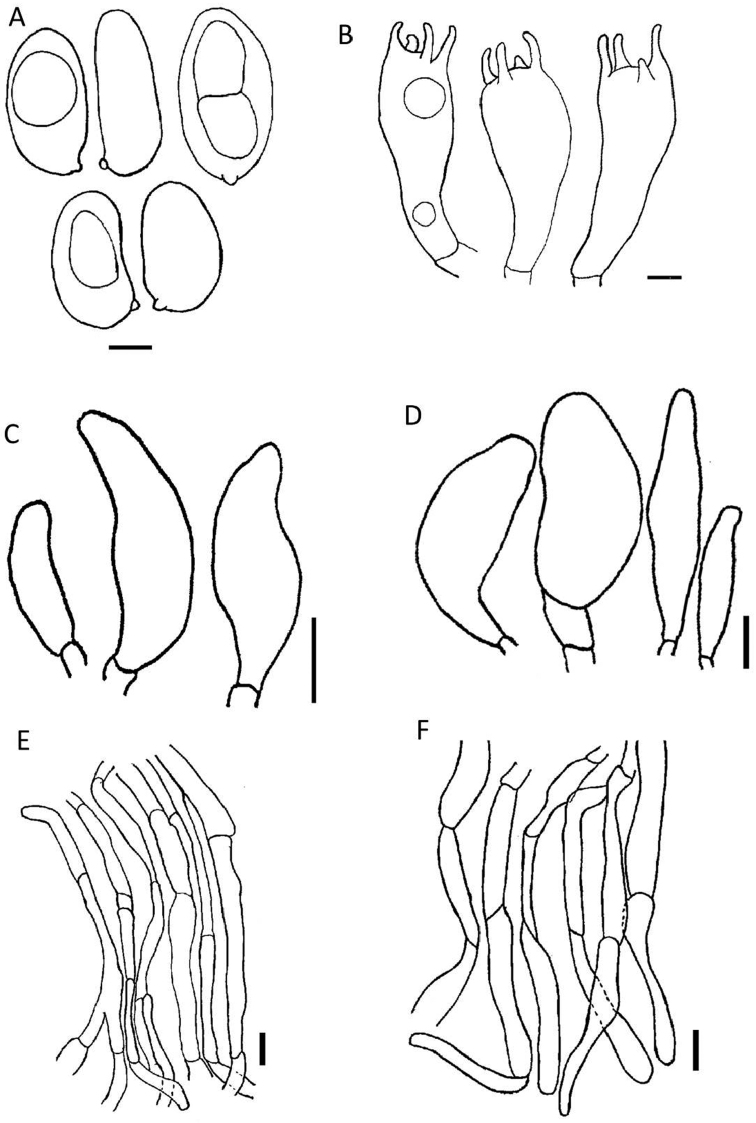
Microscopic structures of *Inosperma bulbomarginatum* (MR00357) **A** basidiospores **B** basidia **C** cheilocystidia **D** caulocystidia **E** pileipellis **F** stipitipellis. Scale bars: 3 μm (**A**); 5 μm (**B**); 10 μm (**C–F**).

**Figure 5.**
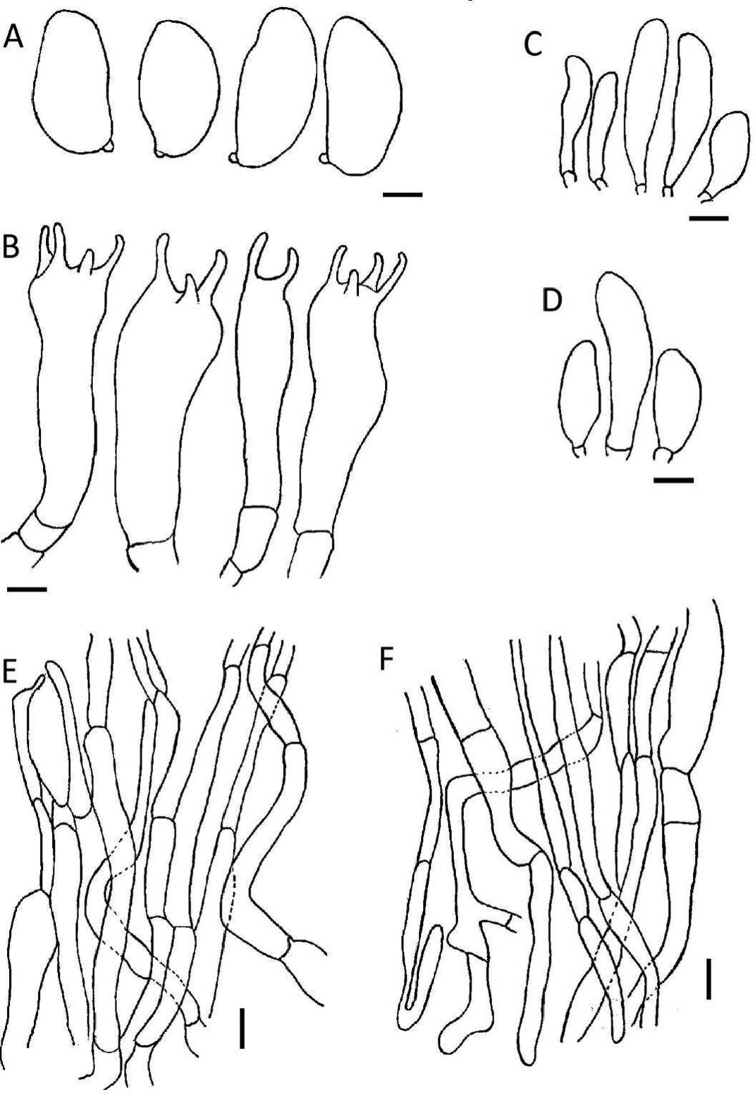
Microscopic structures of *Inosperma flavobrunneum* (HLA0367) **A** basidiospores **B** basidia **C** cheilocystidia **D** caulocystidia **E** pileipellis and **F** stipitipellis. Scale bars: 3 μm(**A**); 4 μm (**B**); 10 μm (**C–F**).

### Taxonomic key to species of *Inosperma* from West Africa

**Table:** 

1	Basidiomata large, pileus 28–38 mm diam., yellow to orange-brown, surface clearly rimose, lamellae adnexed and decurrent, subdistant	***Inosperma flavobrunneum***
–	Basidiomata small, pileus 8.5–15 mm diam., fibrillose, lamellae close	**2**
2	Pileus vinaceous to red, basidiospores 8–10 × 4–7, (sub) globose to cylindrical, sometimes ellipsoid	***I. africanum***
–	Pileus orange-brown to somewhat cinnamon, greyish-white, basidiospores 8–14 × 4–7 μm, elongate	***I. bulbomarginatum***

### New combination

For an evolutionarily-consistent taxonomy, we propose the following combination:

#### Inosperma shawarense

Taxon classificationFungiAgaricalesInocybaceae

(Naseer & Khalid) Aïgnon & Naseer comb. nov.

787A402B-F349-5471-ABAE-C1563CBC8ABD

836296

Inocybe shawarensis Naseer & Khalid, Mycotaxon 132: 912. 2018. Basionym.

##### Notes.

This species is placed in the old *Inosperma* clade which became the genus *Inosperma*, but the combination is not made in the study of Matheny et al. (2020). The new combination is based on molecular phylogenetic data and sequencing the type of *Inocybe shawarensis* (Naseer et al. 2018).

## Discussion

The new species exhibit the overall characteristics often observed in *Inosperma*. These characters include; pileus radially rimose, fibrillose or squamulose and absence of pleurocystidia (Matheny et al. 2020). They can be distinguished from other *Inosperma* species by their remarkable characteristics. In addition, *I. africanum* is common in West Africa and *I. bulbomarginatum* presents a large distribution and was recognised in Zambia in the collections of Bart Buyck (Matheny et al. 2009). However, the low sequence divergences between the sequences (2.2%–2.5%) of ITS and 0.3% of 28S allows us to confirm the wide distribution of *I. bulbomarginatum*.

Phylogenetically, *I. africanum* is nested in *Inosperma* with full support (99% SH-aLRT values, 100% ML Ultrafast bootstrap, 1 BPP) and *I. bulbomarginatum* is indicated as the sister of Old World Tropical clade 1 with full support (100% SH-aLRT values, 100% ML bootstrap, 1 BPP). Sequences of *Inosperma bulbomarginatum* from West Africa and Zambia formed a subclade. *Inosperma flavobrunneum* is nested in Old World Tropical clade 1 and has sister species undescribed in a collection from Zambia, BB3233, G1842 and PC96013. ML and BI analysis, using 28S and RPB2 sequences data, shows most nodes well resolved; for example, the node uniting Old World Tropical clade 2 with the crown group of *Inosperma* is supported with 0.97 BPP, but with weak ML bootstrap as shown in Pradeep et al. (2016); based also on combined data of 28S and RPB2, this node is with weaker support < 50% ML bootstrap.

**Figure 6.**
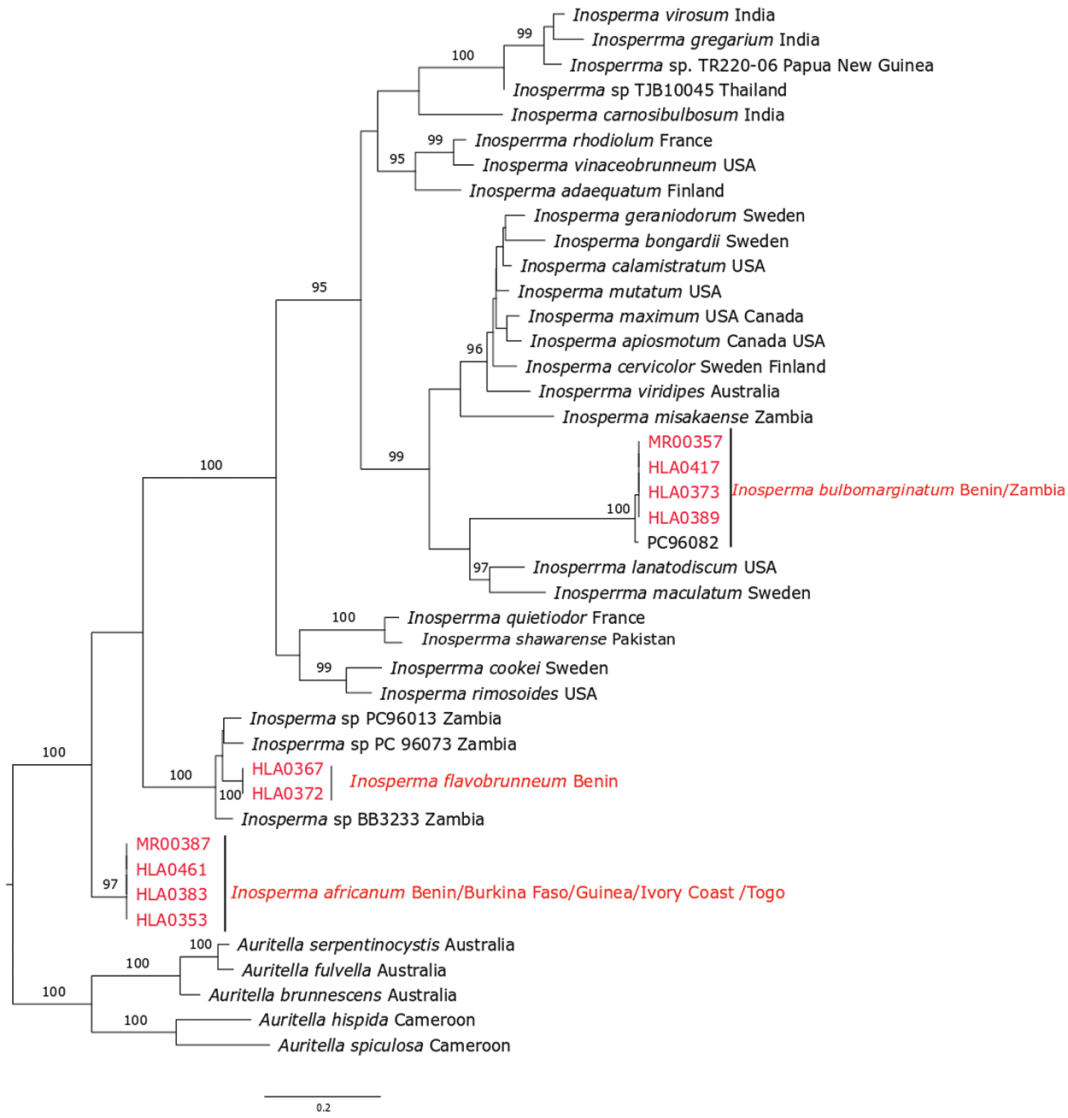
ML phylogeny of *Inosperma africanum*, *I. bulbomarginatum* and *I. flavobrunneum* based on ITS dataset.

The position of each of these new species is confirmed by single data from ITS (Fig. 6). There are several collections from undescribed species in *Inosperma* (e.g. *Inosperma* sp. G1842, *Inosperma* sp. BB3233, *Inosperma* sp. PC 96073, *Inosperma* sp. PC96013, *Inosperma* sp. PC96082, *Inosperma* sp. PC96080 and *Inosperma* sp. Zam07) that are of African origin, thereby attesting the need for further studies of this genus on this continent. Previously, in *Inosperma*, only one species, *Inosperma misakaense*, has been described from Africa before this study (Matheny and Watling 2004). So, this study reinforces the diversity of *Inosperma* in Tropical Africa which now amounts to four described species.

## Supplementary Material

XML Treatment for Inosperma africanum

XML Treatment for Inosperma bulbomarginatum

XML Treatment for Inosperma flavobrunneum

XML Treatment for Inosperma shawarense

## Supplementary materials

Supplementary material 1 Partition for phylogeny analysis Data type phylogeny data https://binary.pensoft.net/file/502369This dataset is made available under the Open Database License (http://opendatacommons.org/licenses/odbl/1.0/). The Open Database License (ODbL) is a license agreement intended to allow users to freely share, modify, and use this Dataset while maintaining this same freedom for others, provided that the original source and author(s) are credited.Hyppolite L. Aïgnon, Sana Jabeen, Arooj Naseer, Nourou S. Yorou, Martin Ryberg
